# Neuronal Activity, Mitogens, and mTOR: Overcoming the Hurdles for the Treatment of Glioblastoma Multiforme

**Published:** 2015-06-01

**Authors:** Kenneth Maiese

**Affiliations:** Cellular and Molecular Signaling, USA

## Abstract

Glioblastoma multiforme (GBM) and other malignant gliomas are considered to be the most prevalent of primary malignant brain tumors. The incidence of these tumors per year is reported as 4.13 per 100,000 individuals per year. The median survival time following the diagnosis of GBM is approximately fifteen months in the setting of providing presently available treatments with surgical resection, radiation, and chemotherapy. Given these statistics, new strategies for the treatment of GBM and other aggressive tumors of the brain are warranted.

In a recent paper in the journal *Cell* [1], Venkatesh *et al.* present new work that elucidates the role of neuronal activity in promoting glioma growth and identifies the mitogen neuroligin-3 as a primary factor to foster the growth of high grade glioma. Neuroligin is a cell adhesion protein that resides on postsynaptic neuronal membranes and maintains synapses between neurons. As a result, neuroligins can control neural networks, neurotransmitter receptors and channels, and also influence new vessel growth in the nervous system. The investigators show that neuroligin-3 was both necessary and sufficient to lead to active high-grade glioma cell proliferation.

Interestingly, in this work neuroligin-3 relied upon the mechanistic target of rapamycin (mTOR) pathway [2]. mTOR is a 289-kDa serine-threonine protein kinase. It oversees gene transcription, stem cell development, cytoskeleton composition, cell metabolism, cell survival, cell senescence, and cell proliferation. mTOR is an important component of the protein complexes mTOR Complex 1 (mTORC1) and mTOR Complex 2 (mTORC2). mTOR also controls pathways of programmed cell death that involve autophagy and apoptosis. In light of the proliferative nature of mTOR, investigations have focused on mTOR as a target to control tumors in the body [2]. In the nervous system, increased activity of mTOR has been associated with neurofibromatosis type 1, tuberous sclerosis, Lhermitte-Duclos disease, and GBM. The United States Food and Drug Administration (FDA) has approved rapamycin (sirolimus) and several rapamycin derivative compounds (“rapalogs”) that inhibit mTOR for the treatment of renal cancer, subependymal giant cell astrocytoma associated with tuberous sclerosis, and neuroendocrine pancreatic tumors.

This new work now identifies that mitogens, such as neuroligin-3, are dependent upon the mTOR pathway and can promote the development of GBM, suggesting that targeting specific mitogens and mTOR may be productive in treating GBM. However, there are several considerations and potential challenges to overcome in developing such treatment strategies. It is important to recognize that tumors can develop resistance to agents that inhibit mTOR signaling. In addition, some tumors may have an increased basal activity of phosphoinositide 3–kinase (PI 3-K), protein kinse B (Akt), and mTOR pathways. Furthermore, blockade of the mTOR pathway can result in the feedback activation of PI 3-K, Akt, and other pathways that can lead to further neoplastic growth. These observations point to the need for the combined inhibition of multiple pathways that involve PI 3-K, Akt, and mTOR. It may be necessary to broaden cellular targets to focus on modulating the PI 3-K–Akt–mTOR axis that has been shown in preclinical studies to increase the sensitivity of radiation against tumor cell growth and the vascular supply of tumors. On the flip side, a fine biological control of mTOR activity also may be required. Extensive inhibition of mTOR activity may lead to memory impairment and cognitive dysfunction such that unacceptable clinical side effects may ensue. The present study provides enormous promise for the treatment of devastating disorders such as GBM, but future work is required to further elucidate the pathways such as mTOR that control tumorigenesis to yield successful clinical outcomes.
